# Defenestration in the Context of Adult Parasomnia: Diagnostic Challenges and Clinical Considerations

**DOI:** 10.1192/j.eurpsy.2025.2331

**Published:** 2025-08-26

**Authors:** O. De Juan Viladegut, H. Andreu Gracia, L. Olivier Mayorga, L. Bueno Sanya, E. Cesari, J. I. Mena, I. Ochandiano, S. Salmerón, L. Pintor

**Affiliations:** 1 Servei de Psiquiatria, Institut Clínic de Neurociències, Barcelona, Spain

## Abstract

**Introduction:**

Abnormal motor and behavioral phenomena during sleep are part of a broader category of sleep behaviour disorder, which may manifest during different stages—either during sleep, wakefulness, or the transitions between these states. Such occurrences are particularly prevalent during early childhood, affecting approximately 15-20% of pediatric populations, while about 4% of adults experience similar events. These sleep disturbances are generally categorized into simple behaviors or more complex behaviors.

**Objectives:**

This case report describes a clinical presentation involving defenestration in the context of parasomnia, with initial concerns about a possible suicide attempt. The objective is to highlight the diagnostic challenges in such cases and emphasize the importance of distinguishing between parasomnia-related behavior and intentional self-harm.

**Methods:**

The patient is a 24-year-old male, born in the US, and currently living in Barcelona as part of a study exchange program. He has been consuming 1SCU of cannabis daily since adolescence. He denies any personal or family psychiatric history but reports experiencing episodes of sleepwalking during his childhood and teenage years.

On presentation, the patient sustained multiple traumatic injuries following an accidental fall from a second-floor window. The event was witnessed by neighbors, who alerted emergency services. The patient has no memories of the event and denies suicidal intent. In the hours preceding the incident, the patient consumed approximately 2SDE of alcohol, but he denies the use of any other substances at the time. Additional testing, including CT of the brain and EEG, revealed no significant abnormalities.

**Results:**

This case presents a diagnostic dilemma, as initial suspicions pointed toward a possible suicide attempt. However, the patient’s history of sleepwalking, especially during childhood, suggests a parasomnia-related etiology. It is crucial to differentiate between childhood-onset sleepwalking, which is often linked to genetic and developmental factors, and sleepwalking that persists or re-emerges in adulthood, which is more strongly associated with psychopathological factors. The persistence of parasomnias in adults may indicate an underlying psychiatric condition.

**Conclusions:**

This case underscores the complexities in diagnosing parasomnias, particularly when severe and potentially dangerous behaviors are involved. While the patient’s history of sleepwalking and lack of psychiatry history suggest a parasomnia-related etiology, the persistence of such behaviors into adulthood warrants careful evaluation for underlying psychopathological factors. Early recognition and accurate diagnosis are paramount to providing effective care and preventing recurrence of such episodes. This case highlights the importance of a multidisciplinary approach, integrating neurology and psychiatry to offer tailored interventions.

**Disclosure of Interest:**

None Declared

